# Supplementary material for: Potassium Effects on NCC Are Attenuated during Inhibition of Cullin E3–Ubiquitin Ligases
Source: Cells. 2021 Dec 29;11(1):95. doi: 10.3390/cells11010095 (PMC8750104; doi:10.3390/cells11010095)
Supplement: Supplementary file 1 [file cells-11-00095-s001.zip › cells-1507874-supplementary.pdf]

SUPPLEMENTAL MATERIAL

Potassium effects on NCC are attenuated during inhibition of Cullin E3-ubiquitin ligases

Supplementary Figure S1. Incubation of ex vivo renal tubule preparations in media with various concentrations of K^+ has no significant effect on total abundance of Cul 1, 3, 4 and 5 or in their n-Cul/Cul ratio. 2

Supplementary Figure S2. The basal neddylation status (activity) of Cul 1, 3, 4 and 5 are different........ 3

Supplementary Figure S3. Treatment of isolated renal tubules ex vivo with MLN4924 had no significant effect on the total abundance of cullins 4

Supplementary Figure S4. MLN4924 effects on NCC and SPAK phosphorylation is intact under different extracellular K+ concentration. 5

Supplementary Figure S5. Inhibition of cullins does not alter K^+ effects on the K^+ channels Kir 4.1 and Kir 5.1 6

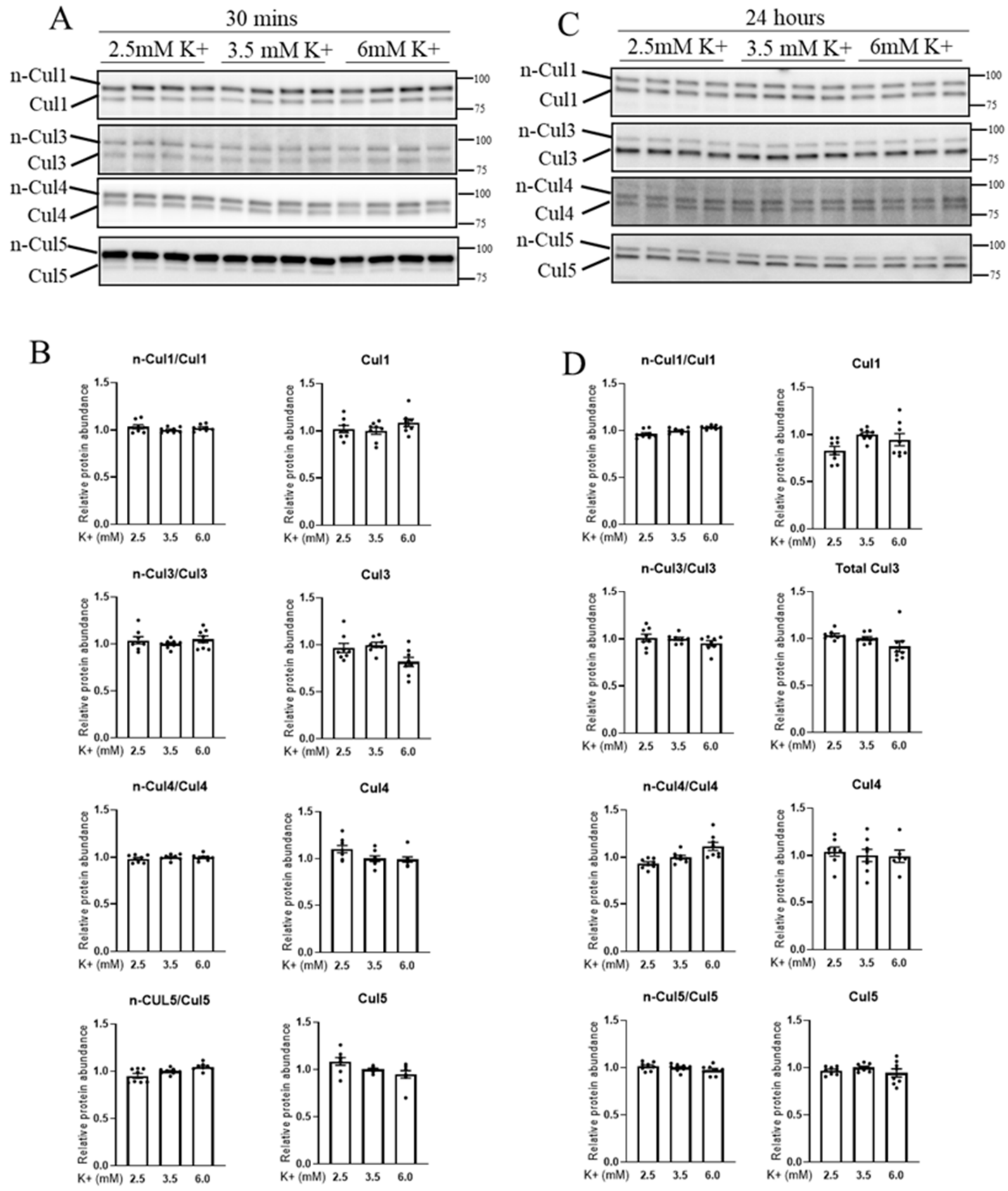

Supplementary Figure S1. Incubation of ex vivo renal tubule preparations in media with various concentrations of K^+ has no significant effect on total abundance of Cul 1, 3, 4 and 5 or in their n-Cul/Cul ratio. A and C) Representative immunoblots of Cul 1, Cul 3, Cul 4 and Cul 5 in isolated renal tubules that were incubated in media containing either 2.5-, 3.5-, or 6 mM K^+ for 30 min or 24 hours, respectively. B and D) Summarized relative protein abundance data from 30 min and 24 hours, respectively. Values are plotted as mean ± SEM with individual values shown (n= 8). For statistical analysis one-way ANOVA followed by the Dunnett's multiple comparison test was used. * indicates $p < 0.05$ relative to 3.5 mM condition.

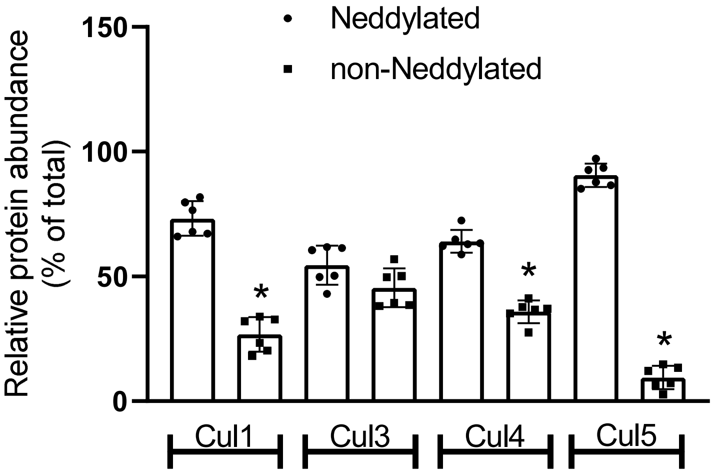

Supplementary Figure S2. The basal neddylation status (activity) of Cul 1, 3, 4 and 5 are different. The relative % of neddylated and non-neddylated Cul 1, Cul 3, Cul 4 and Cul 5 levels in isolated renal tubules are shown. * indicates $p<0.05$ relative to the neddylated group. For statistical analysis, a Student's unpaired t-test was used for individual comparisons of neddylated and non-neddylated Cullins.

A

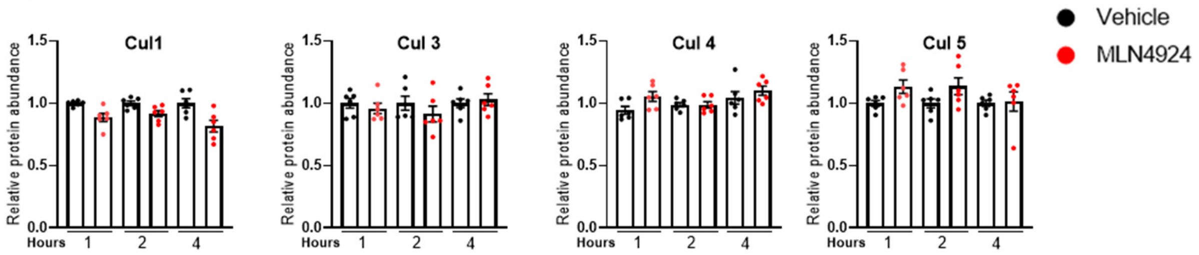

Supplementary Figure S3. Treatment of isolated renal tubules ex vivo with MLN4924 had no significant effect on the total abundance of cullins. A) Summarized data of Cul 1, Cul 3, Cul 4 and Cul 5 abundance in isolated renal tubules that were incubated with either vehicle or the Cullin inhibitor (MLN4924; 0.5 µM) for 1, 2 or 4 h. For statistical analysis, a Student's unpaired t-test was used for individual comparisons of vehicle and MLN4924 treated groups at individual time points.

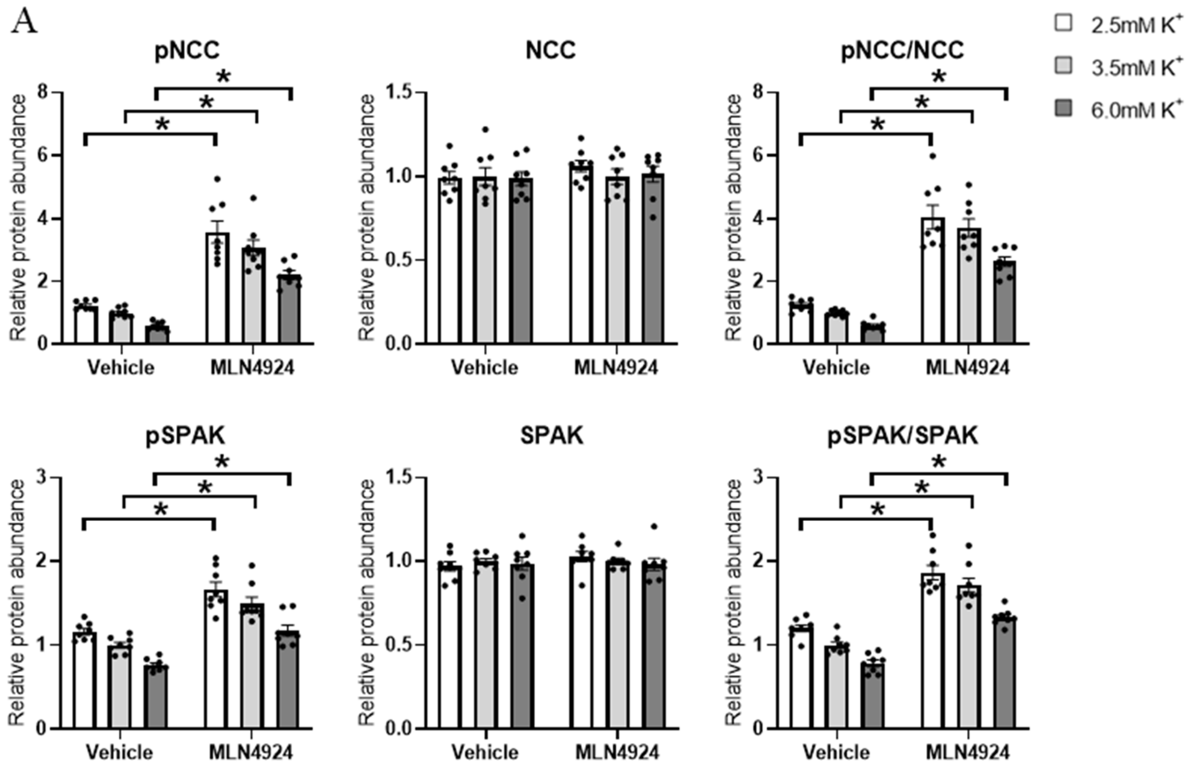

Supplementary Figure S4. MLN4924 effects on NCC and SPAK phosphorylation under different extracellular K+ concentration. A) Summarized data of pNCC, NCC, pNCC/NCC, pSPAK, SPAK and pSPAK/SPAK in isolated renal tubules that were incubated in media containing different K^+ concentrations (2.5 mM, 3.5 mM or 6 mM K^+) in the presence of either vehicle or the Cullin inhibitor (MLN4924; 0.5 μM) for 30 minutes. All data are normalized to vehicle 3.5mM K^+ group. * indicates $p<0.05$ relative to vehicle 3.5mM K^+ group. For statistical analysis Two-way ANOVA followed by the Tukey multiple comparison test was used.

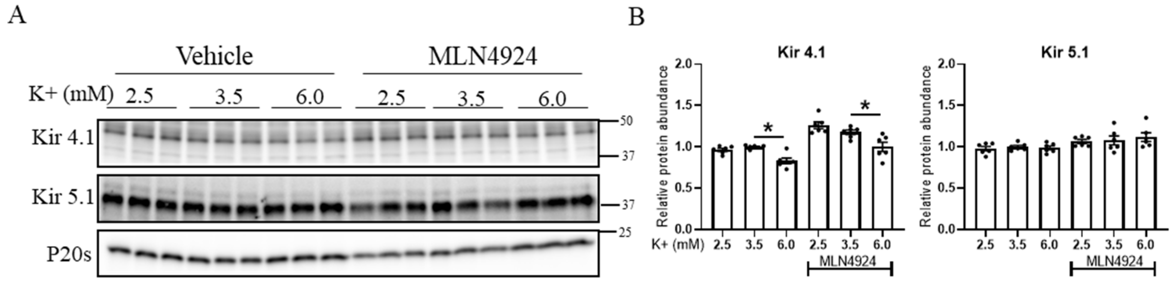

Supplementary Figure S5. Inhibition of cullins does not alter K^+ effects on the K^+ channels Kir 4.1 and Kir 5.1. A) Representative immunoblots and B) summarized data of Kir 4.1 and Kir 5.1 abundance in isolated *ex vivo* renal tubules that were incubated in media containing different K^+ concentrations (2.5 mM, 3.5 mM or 6 mM K^+) in the presence of either vehicle or the Cullin inhibitor (MLN4924; 0.5 μM) for 24 hours. * indicates $p<0.05$ relative to 3.5 mM K^+ in their respective group. For statistical analysis one-way ANOVA followed by the Dunnett's multiple comparison test was used.
